# Hibernating brown bears are protected against atherogenic dyslipidemia

**DOI:** 10.1038/s41598-021-98085-7

**Published:** 2021-09-21

**Authors:** Sylvain Giroud, Isabelle Chery, Mathilde Arrivé, Michel Prost, Julie Zumsteg, Dimitri Heintz, Alina L. Evans, Guillemette Gauquelin-Koch, Jon M. Arnemo, Jon E. Swenson, Etienne Lefai, Fabrice Bertile, Chantal Simon, Stéphane Blanc

**Affiliations:** 1 Research Institute of Wildlife Ecology, Department of Interdisciplinary Life Sciences, University of Veterinary Medicine, Vienna, Savoyenstraße 1, 1160 Vienna, Austria; 2 University of Strasbourg, 4 rue Blaise Pascal, 67081 Strasbourg, France; 3 CNRS, UMR7178, Institut Pluridisciplinaire Hubert Curien (IPHC), 23 rue du Loess, 67087 Strasbourg, France; 4 SPIRAL Laboratories, 21560 Couternon, France; 5 Plant Imaging & Mass Spectrometry (PIMS), Institute of Plant Molecular Biology, CNRS, University of Strasbourg, 12 rue du Général Zimmer, 67084 Strasbourg, France; 6 Department of Forestry and Wildlife Management, Inland Norway University of Applied Sciences, 2480 Koppang, Norway; 7 CNES Paris, 2 Place Maurice Quentin, 75039 Paris Cedex 01, France; 8 Department of Wildlife, Fish and Environmental Studies, Swedish University of Agricultural Sciences, 90183 Umeå, Sweden; 9 Faculty of Environmental Sciences and Natural Resource Management, Norwegian University of Life Sciences, PO Box 5003, 1432 Ås, Norway; 10 University of Auvergne, INRAE, UNH UMR1019, 63122 Saint-Genès Champanelle, France; 11 CARMEN, INSERM U1060/University of Lyon / INRA U1235, Oullins, France

**Keywords:** Fat metabolism, Animal physiology, Dyslipidaemias, Atherosclerosis, Lipoproteins

## Abstract

To investigate mechanisms by which hibernators avoid atherogenic hyperlipidemia during hibernation, we assessed lipoprotein and cholesterol metabolisms of free-ranging Scandinavian brown bears (*Ursus arctos*). In winter- and summer-captured bears, we measured lipoprotein sizes and sub-classes, triglyceride-related plasma-enzyme activities, and muscle lipid composition along with plasma-levels of antioxidant capacities and inflammatory markers. Although hibernating bears increased nearly all lipid levels, a 36%-higher cholesteryl-ester transfer-protein activity allowed to stabilize lipid composition of high-density lipoproteins (HDL). Levels of inflammatory metabolites, *i.e.,* 7-ketocholesterol and 11ß-prostaglandin F2α, declined in winter and correlated inversely with cardioprotective HDL2b-proportions and HDL-sizes that increased during hibernation. Lower muscle-cholesterol concentrations and lecithin-cholesterol acyltransferase activity in winter suggest that hibernating bears tightly controlled peripheral-cholesterol synthesis and/or release. Finally, greater plasma-antioxidant capacities prevented excessive lipid-specific oxidative damages in plasma and muscles of hibernating bears. Hence, the brown bear manages large lipid fluxes during hibernation, without developing adverse atherogenic effects that occur in humans and non-hibernators.

## Introduction

Mammalian hibernation is a seasonal adaptation that allows individuals to survive harsh environmental conditions, including food shortage, during winter (for review, see^1–3^). During the active season prior to winter, hibernating mammals accumulate large fat reserves and nearly double their body (fat) mass from spring to early fall, which would be considered as an obese condition in humans^4–6^. Then, hibernating mammals enter a state of depressed metabolism, known as torpor, which leads to substantial reduction of energy needs and enables individuals to survive the winter^7^. In small (< 8 kg) hibernating species, hibernation corresponds to successive multi-days or -weeks torpor bouts, during which individual metabolic rate (‘MR’) is reduced on average by ~ 95% of basal rates with body temperature lowered below 10 °C, that are interspaced by euthermic phases lasting only few hours^7^. However, some mammalian hibernators of medium (10–20 kg; *e.g.*, *Meles meles*) or large body size (> 20 kg; *e.g.*, *Ursus arctos, Ursus americanus*) or living in tropical and subtropical areas (*e.g.*, *Chirogaleus medius*, *Tenerec ecaudatus*) do not periodically rewarm during winter, and the torpid state hence corresponds to their entire hibernation^8–12^. In particular, bears hibernate at moderate hypothermia (30–36 °C) while lowering their MR by 75% of basal rates during winter^89^. Importantly, most hibernating mammals (so called ‘fat-storing’ hibernators), including bears, do not feed during several months in winter and primarily fuel their energy needs via oxidation of lipids, notably saturated fatty acids (‘SFAs’), mobilized from the white adipose tissue (for review see^13^).

Fat-storing hibernating mammals show marked seasonal changes of lipid metabolism, with drastic increases of all lipid levels during hibernation compared to the summer active period. For instance, increased levels of 1.6 to twofold of non-esterified fatty acids (‘NEFAs’), triacylglycerols (‘TAGs’), and total cholesterol (‘CHT’) were reported in hibernating marmots (*Marmota flaviventris*), golden-mantled ground squirrels (*Callospermophilus lateralis*), thirteen-lined ground squirrels (*Ictidomys tridecemlineatus*), and European badgers (*Meles meles*) during winter^14–18^. Similar seasonal changes were described to occur in the Monito del Monte (*Dromiciops gliroides*), suggesting that marsupials, as eutherians, shift from carbohydrate to lipid-based metabolism during hibernation^19,20^. In particular, cholesterol level rises by almost two-fold in *D. gliroides* during winter compared to summer and spring^19^. Recently, we^18^ have demonstrated that a large hibernator, the brown bear, shows seasonal shifts in its lipid profile similar to those in small hibernators during hibernation in winter. Also, denning American black bears (*Ursus americanus*) showed a significant doubling of NEFA levels in all classes (saturates, monemes and polyenes), along with a 33% increase in albumin, *i.e.,* the plasma fatty acid binding protein, leading to higher NEFA/albumin ratios (4:1) compared to those (3:1) of active black bears in summer^21^. However, despite such seasonal hyperlipidemia and hypercholesterolemia, hibernators do not spontaneously develop pathophysiological syndromes, such as atherosclerosis or other complications linked to lipid peroxidation and oxidative damages during hibernation^22^.

Interestingly, the effect of several months of hibernation on plasma lipid levels resembles that occurring during several days of fast (phase 2 of prolonged fasting) in non-hibernating mammals, *e.g.,* small rodents and rabbits^23–32^, and also in humans^33,34^. When subjected to prolonged fasting, rodents and humans show, however, substantial decreases in TAG and CHT levels, along with increased lipid concentrations in both low-density lipoprotein (‘LDL’) and high-density lipoprotein (‘HDL’) particles, as well as protein and muscle impairments^29,31,32,35,36^ (for review see^37^). In humans, dysfunction of lipoprotein metabolism is associated with hyperlipidemia and hypercholesterolemia, which are direct causes for the development of atheroma plates and ultimately thrombosis (for review see^38^). Moreover, excess lipids, such as cholesterol, are associated with the generation of intimal oxidative stress and inflammatory processes, leading to necrosis, fibrosis, and calcification^39,40^ (for review see^41,42^). In that process, oxysterols, which are oxidized derivatives of cholesterol, and isoprostanes, such as prostaglandin F2 isomers, are known for their pro-inflammatory and pro-oxidative properties and these molecules play key roles in the process of atherogenic dyslipidemia^43,44^ (for reviews see^45,46^).

Instead, hibernators increase seasonally levels of lipoproteins, *e.g.,* very low-density lipoprotein (‘VLDL’) and LDL, responsible for the transport of lipids from the liver to peripheral tissues, during winter, as observed in hibernating golden-mantled ground squirrels^15^ and thirteen-lined ground squirrels^16^. Further, particle size of HDL, involved in the delivery of excess lipids including cholesterol from peripheral tissues to the liver for excretion, increase without any signs of pathological effects in hibernating thirteen-lined ground squirrels^16^. Also, hibernating golden-mantled ground squirrels exhibit significant higher plasma CHT concentration per HDL-cholesterol particles than individuals during the pre-hibernation period^15^. Serum cholesterol and PL (at the exception of phospholipid-ethanolamine) increase in hibernating *vs.* summer active black bears, and variation as large as 650% in total plasma cholesterol along with major changes in lipoproteins occur between early-winter and early-spring, in the European badger^17,47^. Although excess and undesired lipid molecules, including cholesterol, are usually lost from the body through fecal excretion or via conversion into bile acids, lipids can also be re-esterified via a futile cycle between HDL and VLDL/LDL within the organism. To date, most of the studies on lipid trafficking and lipoprotein dynamics during hibernation have been conducted in small hibernators, which typically rewarm and reverse metabolism at regular intervals during hibernation. However, little is known to that respect in large species, such as bears, which do not eat, drink, urinate, defecate or exhibit arousal episodes^48,49^, hence cannot eliminate excess cholesterol, during hibernation. Therefore, one would expect unique physiological and biochemical adjustments of cholesterol and lipoprotein metabolisms in hibernating bears during winter.

Our study aimed at investigating the lipoprotein and cholesterol metabolisms in free-ranging Scandinavian brown bears captured both in the summer active season and during their six to seven months of hibernation. For this purpose, we performed analyses of lipoprotein composition and assessed activities of key enzymes of lipid metabolism in blood plasma (to assess lipid trafficking and cycles) and determined lipid composition in skeletal muscles (reflecting cholesterol utilization). We further measured the levels of oxysterols and isoprostanes (prostaglandin F2 isomers). Hibernating brown bears selectively retain unsaturated fatty acids, which are more prone to peroxidation than saturated lipids with less unsaturation, in their tissues during winter^18^. Hence, we also determined plasma antioxidant capacities and reserves along with oxidative damages in plasma and muscle to determine possible oxidative implications linked to changes in lipid and lipoprotein metabolisms during hibernation. Specifically, we hypothesized that bears manage the large fluxes of lipids and sterols during their hibernation by increasing lipoprotein lipid concentrations, allowing the activation of futile cycles of re-esterification via lipoprotein metabolism.

## Material and methods

### Study area

The field of study encompassed about 21,000 km^2^ in south-central Sweden (61° N, 15° E). Fieldwork was carried out in Dalarna County during February (winter) and in June (summer) 2011, 2012 and 2013. The topography in this region is rolling hills, with < 10% above 750 m above sea level. The area is forested and dominated by Scots pines (*Pinus sylvestris* L.) and Norway spruces (*Picea abies* H. Karst). The region is sparsely populated, but comprises an extensive network of forestry tracks and some paved roads, and is used by hunters with dogs, not only during the moose (*Alces alces*) hunting season in September and October, but also during the bear hunting season. The estimate of total population for brown bears in Sweden was 2,757 (95% credible interval: 2,636 to 2,877) individuals in 2018^50^. The hunting period for bears begins on 21 August and ends when the area-specific quota has been filled, usually mid- to late September^51^, and can overlap with the pre-denning period^10^. Most den abandonments occur early in the denning season: a recent study documented that 22% of bears changed dens during winter, but only 4% after mid-December^52^.

### Animals and sample collection

All personnel in the Scandinavian Brown Bear Research Project (SBBRP) have advanced experience and training in capturing and handling free-living brown bears during all seasons. Brown bears are captured annually by the SBBRP and fitted with neck collars, which include a global positioning system (‘GPS’), dual-axis motion sensors (to monitor activity), very-high-frequency (‘VHF’) transmitters, and a global system for mobile mobilization (‘GSM’) modem (Vectronic Aerospace GmbH, Berlin, Germany). As a backup to relocate bears if the collar malfunctioned, VHF transmitters were implanted into the abdomen (Telonics, Inc, Mesa, Arizona, USA)^53^. GPS positions are recorded every 30 min to 1 h. Bears that were the offspring of marked females were followed from birth; otherwise, age was determined by counting the annuli of a cross-section of the premolar roots^54^.

Fourteen bears, including two animals studied during two consecutive years (for more details see Table 1), were included in this study. During winter, bears hibernate at moderate (30–36 °C) hypothermia^9^, and the torpid state hence corresponds to their entire hibernation from November to April^10^. Body temperature measurements of the bears were performed during hibernation in winter and during the active phase in summer (see Table 1 for details). During hibernation, torpid animals were captured in February 2011, 2012, and 2013 by darting them directly in their den, as previously described^55^. The same individuals were re-captured when active in June 2011, 2012, and 2013 by darting from a helicopter^56^. In winter, the anesthesia consisted of a combination of medetomidine-ketamine-tiletamine-zolazepam^10,55^, whereas bears in summer were anesthetized with a mix of medetomidine-tiletamine-zolazepam^53^. Once anesthetized, we took each bear out of the hibernating den in winter or located it on the ground in the case of darted individuals from a helicopter in summer, and placed it on an insulated blanket to conduct the experiments and to collect tissue samples. The same tissues, *i.e.,* blood (~ 20–30 mL) and skeletal muscle (*Vastus lateralis*) biopsies (~ 250–350 mg), were taken from these bears during both seasons and were used to perform the biochemical analyses. Blood samples were collected, approximately 20–30 min after darting, from the jugular vein in heparinized tubes (BD Vacutainer, Lithium Heparin 34 IU, Plus Blood Collection Tubes 2.0 mL), then plasma was isolated by centrifugation within one hour at 3,500 rpm at 4 °C for 10 min. Plasma and muscle samples were snap-frozen and stored at − 80 °C for subsequent analyses.

**Table 1:** Summary of the brown bears used in the study and their individual physiological parameters.

ID	Sex	Year	Body mass (kg)		Body temperature (°C)		Tissues		Analyses
			Summer	Winter	Summer	Winter	Summer	Winter	
0825	F	2011	47.0	58.0	40.5	34.7	P M	P	LM/IF
0904	F	2011	72.0	57.0	37.3	34.1	P M	P M	LM/IF
0908	M	2011	51.0	58.0	39.9	33.4	P M	P M	LM/IF
1004	M	2011	22.0	21.0	39.2	< 32.0	P	P M	LM/IF
1011	F	2012	59.0	56.0	40.8	34.2	P M	P M	LP/AR/IF
1015	M	2011	27.0	25.0	38.6	33.1	–	P	LM/LP/AR/IF
1017	F	2011	28.0	35.0	39.2	36.2	P M	P M	LM/IF
1017	F	2012	55.5	56.3	39.3	35.8	P	–	LP/AR/IF
1104	F	2012	29.0	30.2	39.4	< 32.0	P	P	LP/AR/IF
1104	F	2013	57.0	52.0	39.4	32.1	P	P M	LP/AR/OS/IF
1105	F	2013	60.0	55.0	39.4	< 32.0	P	P M	LP/AR/OS/IF
1110	F	2013	58.0	53.0	40.0	35.1	P M	P M	LP/AR/OS/IF
1202	M	2013	48.0	48.0	41.0	33.5	P M	P M	LP/AR/OS/IF
1204	M	2013	38.0	40.0	40.9	32.2	P M	P M	LP/AR/OS/IF
1207	M	2013	64.5	54.0	39.1	32.7	P M	P M	LP/AR/OS/IF
1209	F	2013	27.0	30.0	40.7	32.9	P M	P M	LP/AR/OS/IF

We applied linear mixed-effects models to test for the effect of season (fixed variable) on the different lipoproteins, lipid groups, enzymatic activities, inflammatory processes, antiradical resistance, and oxidative stress markers (predicted variables) in each tissue, *i.e.* muscle tissue (‘M’), and blood plasma (‘P’). Bear’s ID was included as random effect for taking repeated measurements among animals into account.

*LP* Lipoproteins, *LM* Lipidomics, *AR* Blood Antiradical Resistance (KRL Test), *IF* Inflammatory Processes, *OS* Oxidative Stress.

### Ethical statement

All captures and subsequent interventions carried out on the animals were approved by the Ethical Committee on Animal Experiments, Uppsala, Sweden (applications Dnr C3/2016 and Dnr C18/2015), the Swedish Environmental Protection Agency (NV-0741-18), and the Swedish Board of Agriculture (Dnr 5.2.18-3060/17). Furthermore, all experiments were carried out in compliance with the ARRIVE guidelines (https://arriveguidelines.org), and all methods were performed in accordance with relevant guidelines and regulations.

### Overview of biochemical analyses

To assess lipid transport within the organism, we performed quantification of the lipid composition of main categories of plasma lipoproteins, *i.e.*, VLDL, LDL, intermediate-density lipoprotein (‘IDL’) and HDL, and further quantified the different HDL sub-fractions via Size Exclusion Chromatography (SEC) with online enzymatic detection of lipoprotein components^57^ and electrophoresis using non-denaturing polyacrylamide gradient gels^58–60^, respectively (read the sections ‘Determination of plasma and lipoprotein lipid composition’ and ‘Electrophoretic separation of high-density lipoprotein sub-fractions’ in supplementary methods for more details). Hence, for each lipoprotein, we identified and quantified the main groups of composing lipids: non-esterified cholesterol (‘CHNE’), cholesteryl-esters (‘CE’), CHT, free cholesterol (‘CHF’), NEFA, phospholipids (‘PL’), and TAG. We also quantified the same lipid molecules, along with the concentration of ß-hydroxybutyrate (‘ßOHB’) using specific kits, in blood plasma. We additionally determined plasma enzymatic activities of cholesteryl-ester transfer protein (‘CETP’), lecithin-cholesterol acyltransferase (‘LCAT’), and phospholipid transfer protein (‘PLTP’) via the use of commercial enzymatic kits (see supplementary methods for further details). Using mass spectrometry, we further assessed pro-inflammatory and pro-oxidative properties of the blood by measuring plasma levels of three oxysterols, *i.e.*, 7-ketocholesterol, 7α-hydroxycholesterol and 7ß-hydroxycholesterol, and four isoprostanes, *i.e.*, 8-iso prostaglandin F2α (8-iso PGF2α), 8-iso-15(R) prostaglandin F2α (8-iso -15(R) PGF2α), 11ß-prostaglandin F2α (11ß-PGF2α), 15(R)-prostaglandin F2α (15(R)-PGF2α) (read supplementary methods for further details). Also, we assessed the antiradical resistance of the blood, *i.e.*, the half-time of the hemolysis of red blood cells (‘HT50’) exposed to a controlled free radical attack, and various antiradical defense reserves (‘RESEDA-1’, ‘RESEDA-2’, ‘RESEDA-3’) within plasma by using the biological KRL/RESEDA™ test from Kirial International/Laboratories Spiral, Couternon, France^61–64^ (see supplementary methods for further details). Finally, we measured plasma and muscle levels of oxidative stress markers, malondialdhyde (MDA)-protein adducts and protein-carbonyls via the use of commercial ELISA kits (see supplementary methods for further details).

### Statistical analyses

Data analyses were performed using R 3.4.4^65^. We used linear mixed-effects models (‘lme’ in library ‘nlme’^66^) to test for the effect of season (fixed variable) on the various predicted variables, considering repeated measurements among animals by including bear’s ID as random effect, with a compound symmetry (CS) as a structure for covariance (equivalent to paired Student’s t-test). Standardized residuals from statistical models were tested for normality using Kolmogorov–Smirnov tests, and data were either log- or Box-Cox transformed when necessary. Predicted variables included (i) the different lipoprotein categories, sub-classes or ratios, (ii) the different lipid groups, (iii) the plasma enzymatic activities, (iv) the concentrations of oxysterols and isoprostanes, (v) the antiradical resistance of red blood cells or the various antiradical defense reserves, and (vi) the plasma levels of oxidative damages. Initial inspection of the data gave no evidence for an effect of sex or sampling year on any of predicted variables. Differences of means between seasons were assessed. Values are means ± SE and means of log- or Box-Cox transformed differences ± SE (except for percentages of total neutral lipids, which are actual log-transformed differences). We further performed regression models between levels of 7-Ketocholesterol or 11ß-PGF2α and proportions of HDL 12–9 nm, HDL3c and HDL2b particles by using similar linear mixed-effects with bear’s ID as random effect to consider repeated measures. We also applied linear mixed-effects models to test for potential associations between the antiradical resistance of red blood cells and the specific markers of either lipid peroxidation (MDA-protein adducts) or protein oxidation (protein carbonyls) in muscle. Further, we computed (conditional) R-squared values accounting for the random effect in linear mixed-effects models (‘r.squaredGLMM’ in library ‘MuMIn’^67^). P-values < 0.05 were considered as significant.

## Results

### Plasma and muscle lipid levels

**Figure 1:**
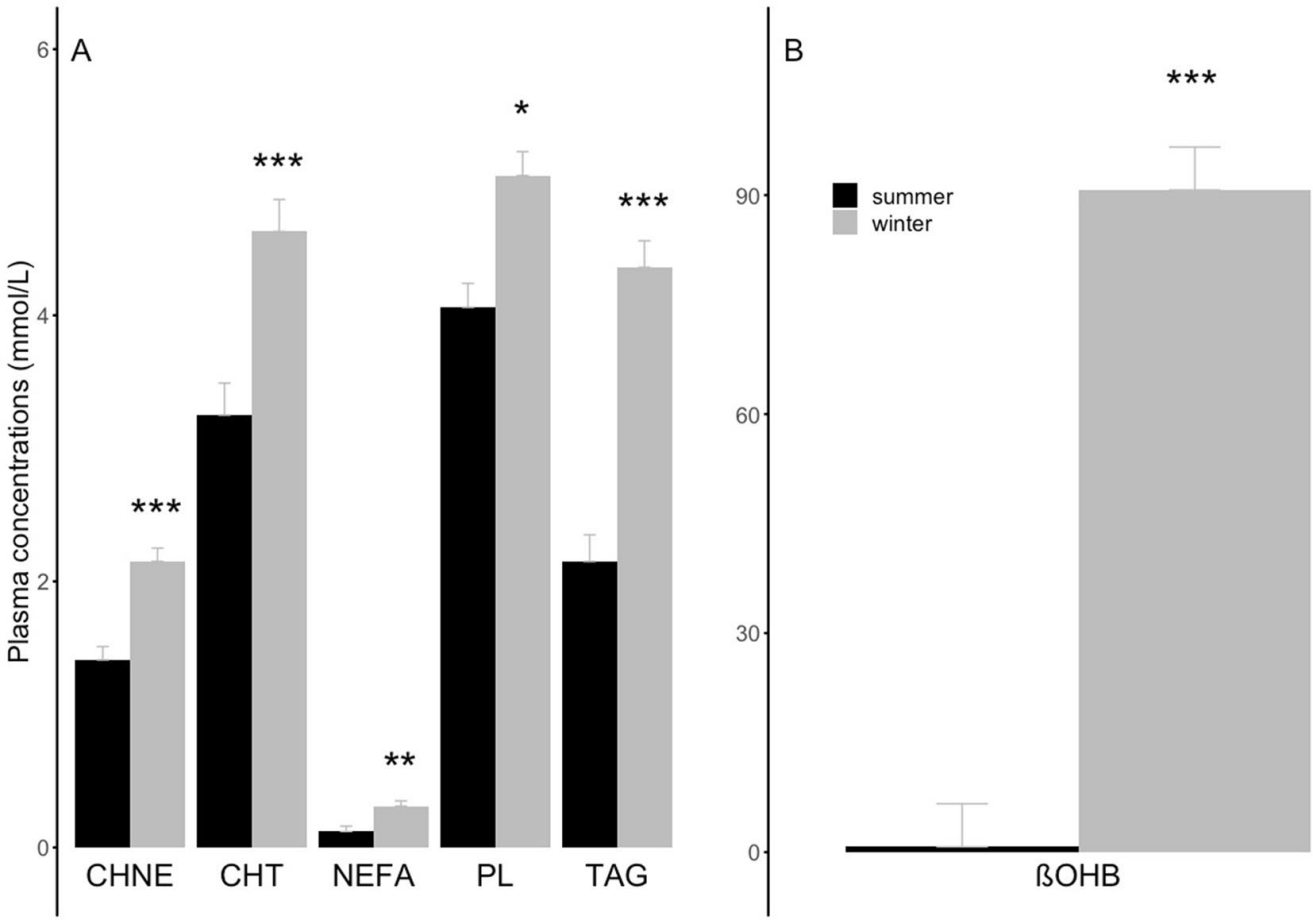
Plasma concentrations of (**A**) the main categories of lipids and (**B**) ß-hydroxybutyrate (‘ßOHB’) from summer active (‘summer’) and winter hibernating (‘winter’) brown bears. In contrast to summer active individuals, winter hibernating bears were all torpid as indicated by their body temperature during hibernation (see Table 1). The lipid main categories are non-esterified cholesterol (‘CHNE’), total cholesterol (‘CHT’), non-esterified fatty acids (‘NEFA’), phospholipids (‘PL’), and triacylglycerols (‘TAG’). Error bars represent standard errors. Winter levels differing significantly from their respective summer level are denoted by subscripts (*p < 0.05, **p < 0.01, ***p < 0.001).

Plasma concentrations of the main lipid categories and molecules were higher in hibernating brown bears than summer-active individuals. Specifically, we found significant higher plasma levels of CHNEs (53% increase), CHT (42% increase), NEFAs (67% increase), PLs (24% increase), and TAGs (twofold increase) during hibernation (Fig. 1A, Supplementary Table S1). In addition, plasma concentration of ßOHB was tremendously higher, by 120-fold, in brown bears during winter hibernation than the summer active season (Fig. 1B, Supplementary Table S1).

**Figure 2:**
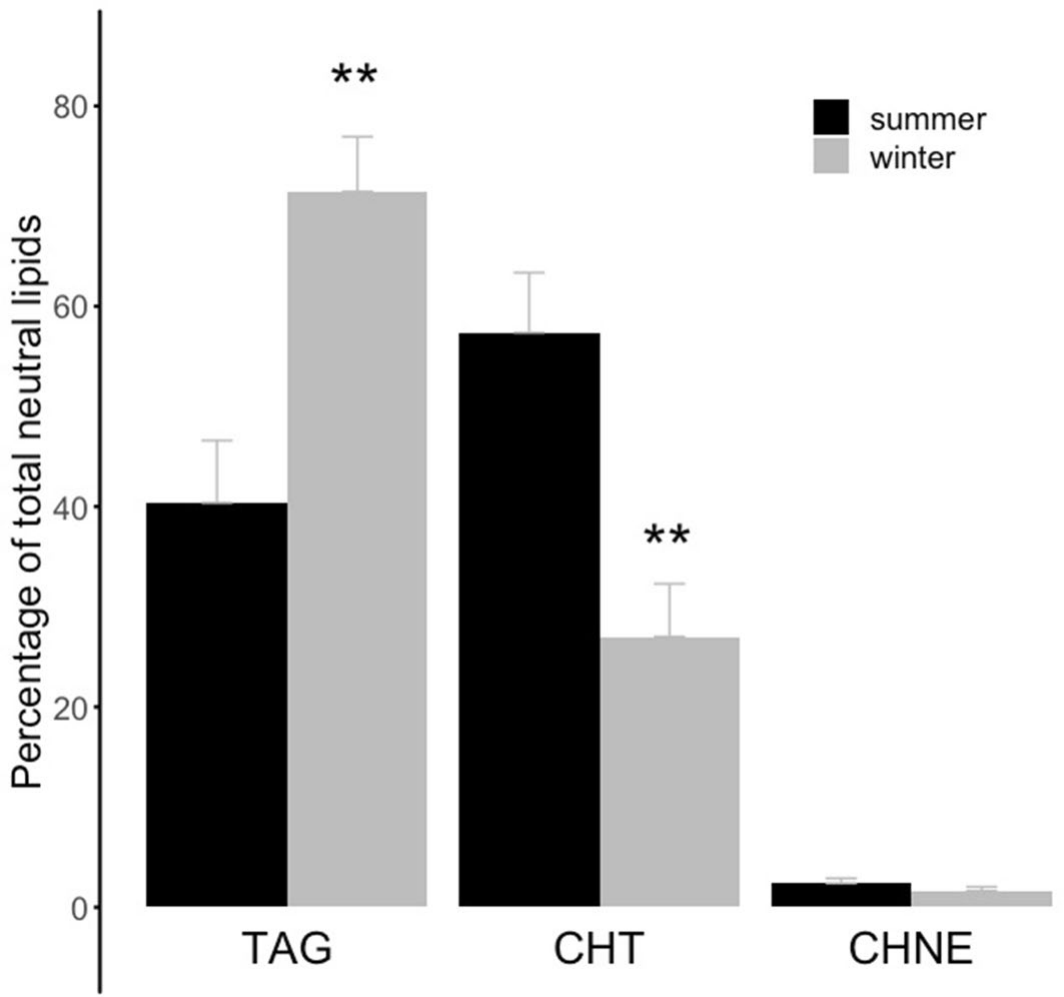
Muscle levels of neutral lipids according to season. Proportions - % of total neutral lipids - of triacylglycerols (‘TAG’), total cholesterol (‘CHT’), and non-esterified cholesterol (‘CHNE’) in skeletal muscle of summer active (‘summer’) and winter hibernating (‘winter’) brown bears. In contrast to summer active individuals, winter hibernating bears were all torpid as indicated by their body temperature during hibernation (see Table 1). Error bars represent standard errors. Winter levels differing significantly from their respective summer level are denoted by a subscript (**p < 0.01).

Muscle concentrations of neutral lipids were also different in brown bears during winter hibernation compared to the active state in summer. Hibernating bear muscles showed a 77% higher level of TAGs and a 2.1-fold lower level of CHT compared to muscles from summer active individuals (Fig. 2, Supplementary Table S1). No significant seasonal change was detected in the level of CHNEs in muscle (Fig. 2).

### Lipoprotein composition, subclasses and sizes

**Figure 3:**
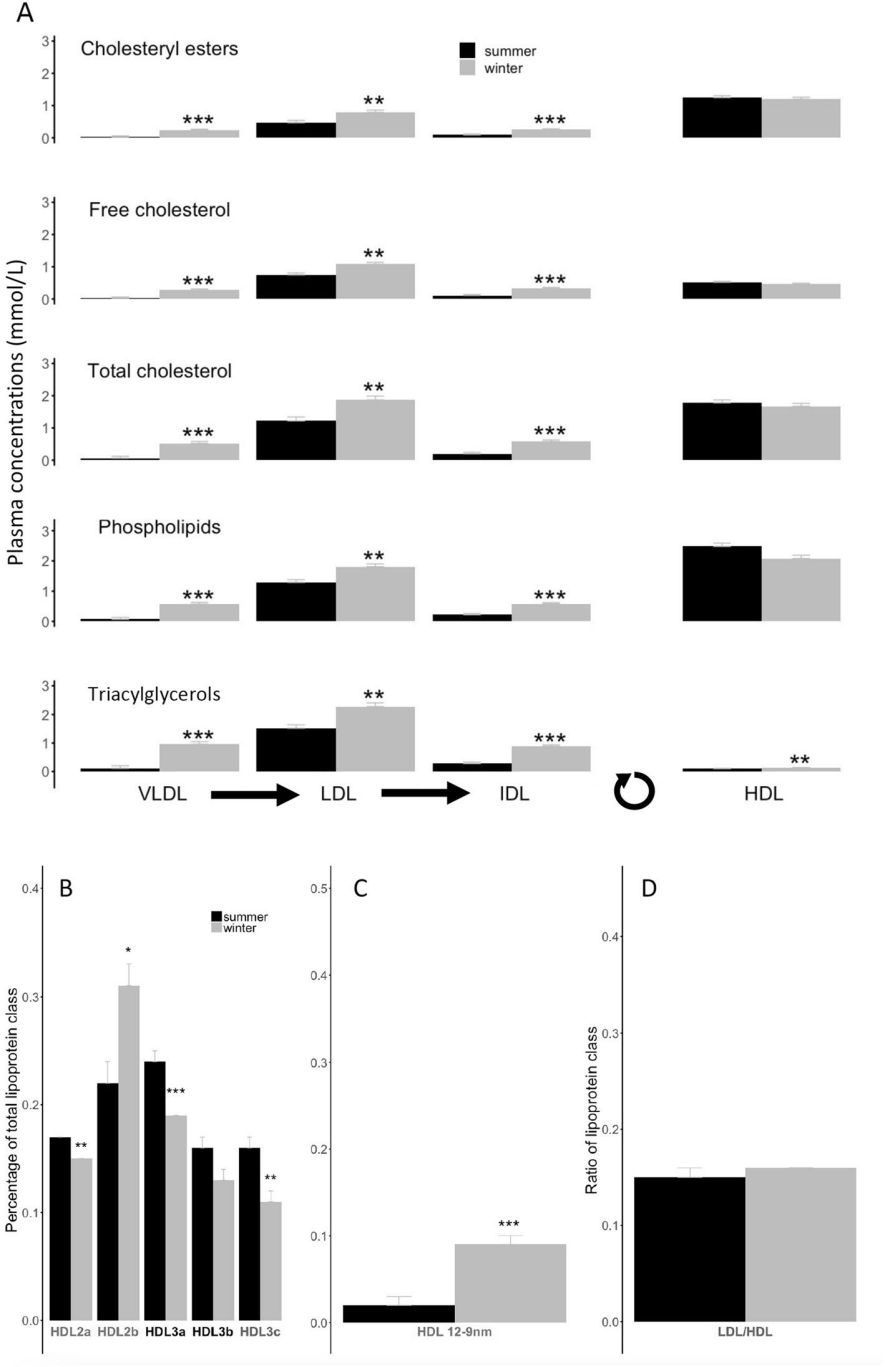
Lipoprotein compositions, subclasses and sizes according to season. The panel (**A**) presents the lipid compositions of the different lipoprotein classes which include very low-density lipoproteins (‘VLDL’), low-density lipoproteins (‘LDL’), intermediate-density lipoproteins (‘IDL’), and high-density lipoproteins (‘HDL’). Black arrows between lipoproteins indicates the formation pathway from VLDL to IDL, and the circular arrow represents the lipid cycling from peripheral tissues leading to HDL formation. Concentrations of the different lipid groups, i.e., cholesteryl esters, free cholesterol, total cholesterol, phospholipids and triacylglycerols, were determined for each of the lipoprotein classes from summer active (‘summer’) and winter hibernating (‘winter’) brown bears. The lower panels show the different proportions - % of total lipoprotein class - of (**B**) high-density lipoproteins 2a (‘HDL2a’), 2b (‘HDL2b’), 3a (‘HDL3a’), 3b (‘HDL3b’), and 3c (‘HDL3c’), and (**C**) high-density lipoprotein subunits with a diameter greater than 12.9 nm (‘HDL 12-9 nm’) that were determined in blood plasma from summer active (‘summer’) and winter hibernating (‘winter’) brown bears. (**D**) The ratio between low-density and high-density lipoproteins (‘LDL/HDL’) is further indicated. Lipoprotein particles associated with anti-atherogenic effects are indicated in green, whereas pro-atherogenic factors are indicated in red-Bordeaux. In contrast to summer active individuals, winter hibernating bears were all torpid as indicated by their body temperature during hibernation (see Table 1). Error bars represent standard errors. Winter levels differing significantly from their respective summer level are denoted by subscripts (*p < 0.05, **p < 0.01, ***p < 0.001).

Composition of lipoproteins was substantially different in brown bears during winter hibernation compared to the summer active phenotype. We found significant increases of TAG levels by 9, 1.5, 3.2, and 1.4-fold within all lipoprotein classes, namely VLDL, LDL, IDL, and HDL respectively, from winter hibernating bears compared to summer active animals (Fig. 3A, Supplementary Table S2). Further, concentrations of CEs, CHF, CHT, and PLs increased by 8.8, 1.5, 2.8-fold on average in VLDL, LDL, and IDL respectively, but no significant changes were detected in the HDL subunit between winter hibernating and summer active bears (Fig. 3A, Supplementary Table S2).

Similar to lipoprotein composition, subclass proportions and sizes of lipoproteins were also altered, although in different ways between classes/sizes, in hibernating brown bears during winter compared to active individuals in summer. Plasma proportions of all subclasses of HDL decreased significantly by 20%, except HDL2b, which is known to be cardioprotective, that increased by 40% in hibernating bears versus summer active animals (Fig. 3B, Supplementary Table S3). Further, we observed a higher plasma proportion of large-size HDL particles, *i.e.,* those with a diameter greater than 12.9 nm, in brown bears during winter hibernation than during the summer active period (Fig. 3C, Supplementary Table S3). No significant seasonal change was detected in the ratio between LDL and HDL (LDL/HDL) (Fig. 3D, Supplementary Table S3).

### Enzymatic activity levels

**Figure 4:**
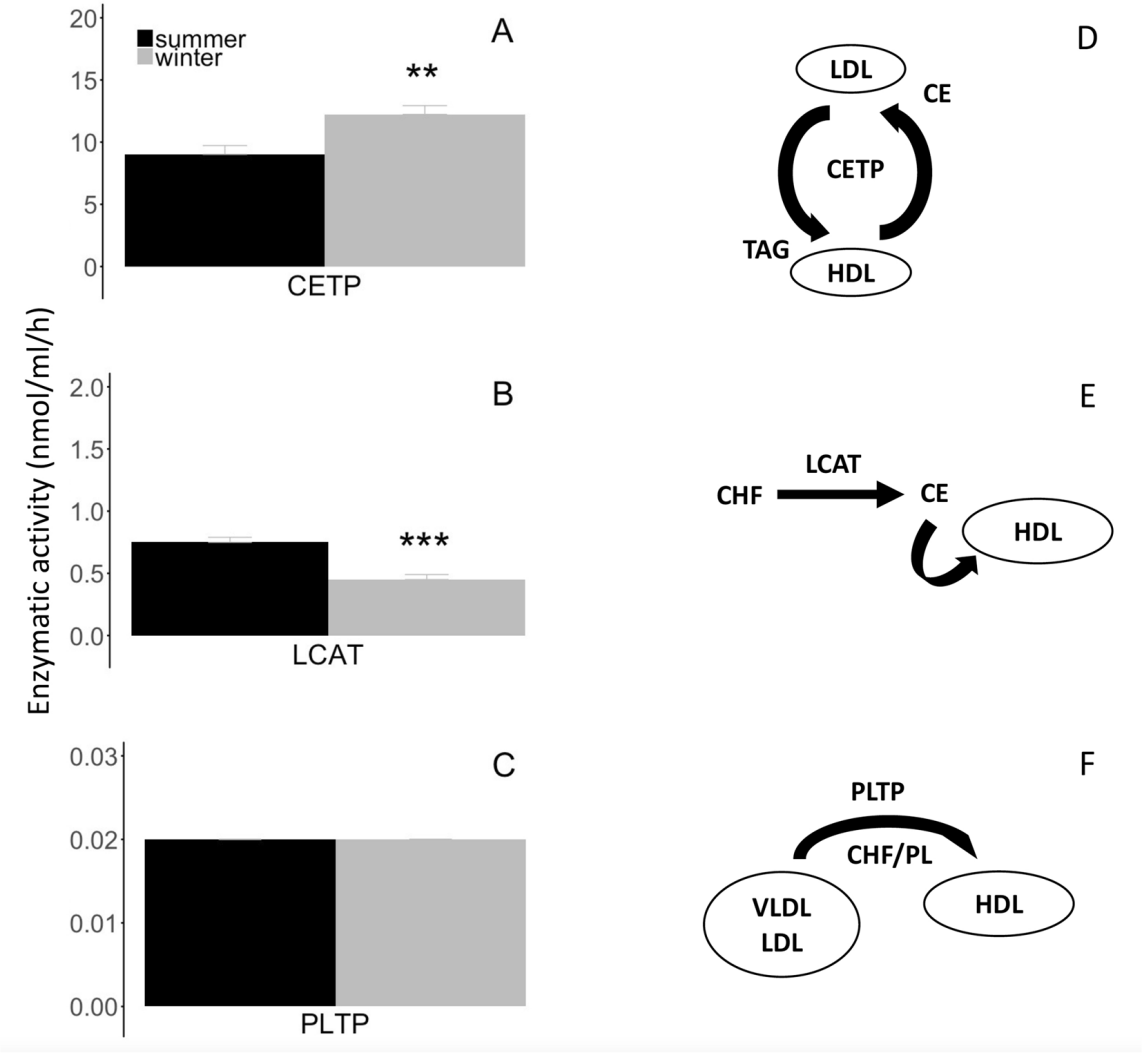
Activities of plasma phospholipids and cholesteryl-esters transfer enzymes according to season. Enzymatic activities of (**A**) cholesteryl ester transfer protein (‘CETP’), (**B**) lecithin-cholesterol acyltransferase (‘LCAT’), and (**C**) phospholipid transfer protein (‘PLTP’) were measured in blood plasma from summer active (‘summer’) and winter hibernating (‘winter’) brown bears. The specific reaction catalyzed by each of the enzymes is depicted of the right side inside from each graph. (**D**) CETP transfers cholesteryl-esters (‘CE’) from high-density lipoprotein (‘HDL’) to low-density lipoprotein (‘LDL’) in exchange to triacylglycerols (‘TAG’). (**E**) LCAT converts free cholesterol (‘CHF’) into CE, which is then sequestered in HDL. (**F**) PLTP mediates the transfer of CHF and phospholipids (‘PL’) from very low-density lipoprotein (‘VLDL’) and LDL into HDL. In contrast to summer active individuals, winter hibernating bears were all torpid as indicated by their body temperature during hibernation (see Table 1). Error bars represent standard errors. Winter levels differing significantly from their respective summer level are denoted by subscripts (**p < 0.01, ***p < 0.001).

Activities of enzymes involved in lipid and lipoprotein metabolisms were substantially modified in hibernating bears during winter compared to active individuals in summer. We found a significant 36% increase in the activity of plasma CETP and a significant 40% reduction of the activity of plasma LCAT in hibernating brown bears versus summer active animals (Fig. 4A,B, Supplementary Table S4). No significant seasonal change was detected for the activity of PLTP (Fig. 4C,F, Supplementary Table S4).

### Oxysterols and isoprostanes

Among the different oxysterols and isoprostanes for which MRM assays were developed, 7ß-hydroxycholesterol, 8-iso PGF2α, 8-iso-15(R) PGF2α, and 15(R)-PGF2α remained below the limit of detection in all samples. The limit of detection was also not reached for 7-ketocholesterol in 7 of 16 winter samples, and for 7α-hydroxycholesterol in 8 of 16 summer samples and in all winter samples but one. On the other hand, 11ß-PGF2α was nicely detected in all samples.

**Figure 5:**
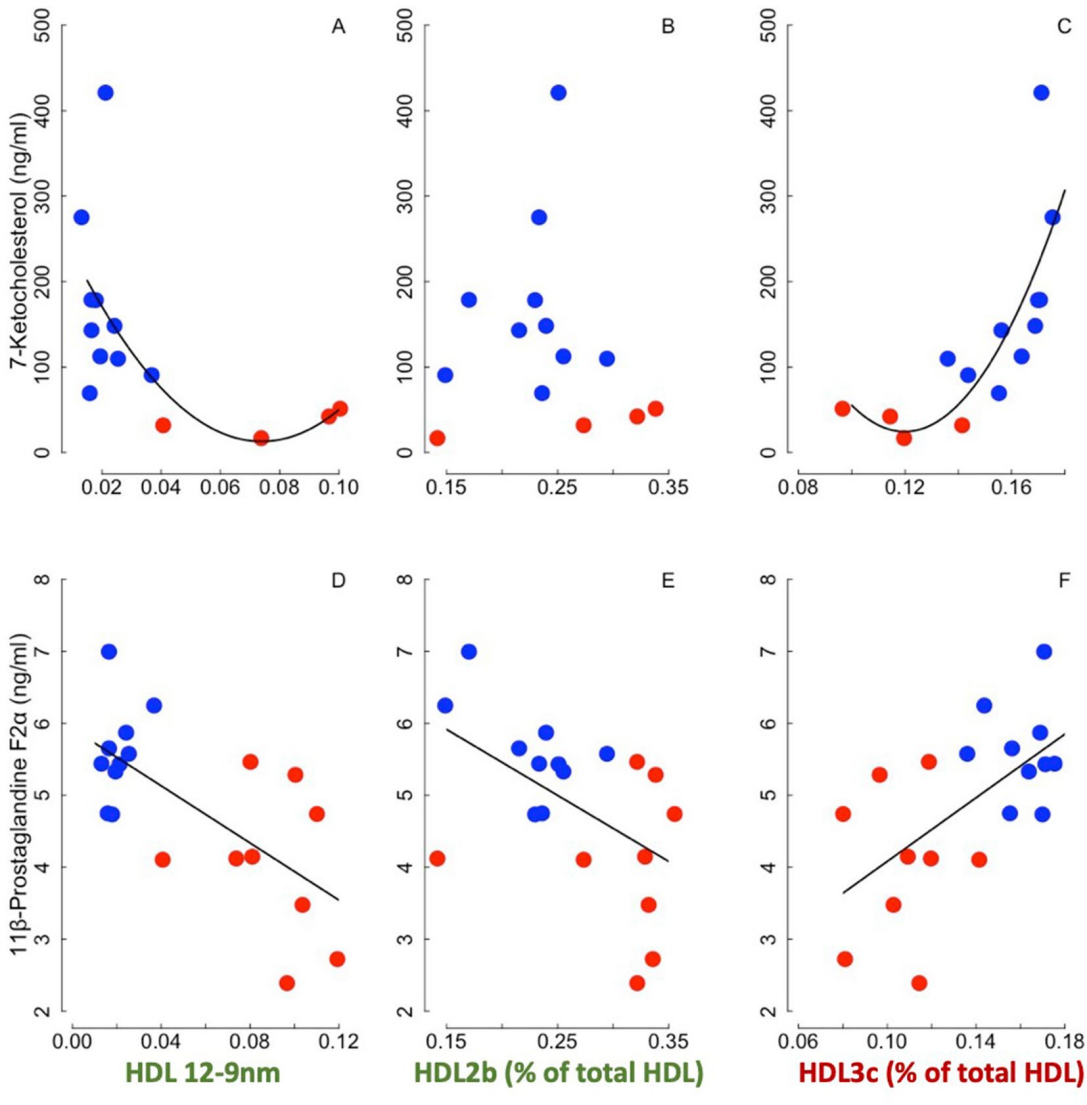
Oxysterol and isoprostane as a function of high-density lipoprotein subunits and size. Levels of the oxysterol ‘7-Ketocholesterol’ and of the isoprostane ‘11ß-Prostaglandin F2α’ are represented as functions of proportions of (A-D) HDL subunits of diameter greater than 12.9 nm (‘HDL 12.9 nm’), (B-E) high-density lipoprotein 2b (‘HDL2b’), or (C-F) high-density lipoprotein 3c (‘HDL3c’) in summer active (blue dots) and winter hibernating (red dots) brown bears. Please note the exponential relationships between levels of 7-Ketocholesterol and (A) HDL 12.9 nm or (C) small HDL3c subunits, indicating a strong effect of intermediate and large HDL particle size in reducing levels of cholesterol derivatives with inflammatory properties. Lipoprotein particles associated with anti-atherogenic effects are indicated in green, whereas the pro-atherogenic factor (HDL3c) is indicated in red-Bordeaux. Regression statistics from linear mixed-effects models: (**A**) R^2^ = 0.34, t = 6.14, p < 0.001; (**B**) R^2^ = 0.04, t = − 1.04, p = 0.41; (**C**) R^2^ = 0.58, t = 16.84, p = 0.03; (**D**) R^2^ = 0.43, t = − 3.68, p = 0.008; (**E**) R^2^ = 0.26, t = − 2.52, p = 0.04; (**F**) R^2^ = 0.36, t = 3.16, p = 0.02.

We found significantly lower plasma concentrations of both the oxysterol, 7-ketocholesterol, and the isoprostane, 11ß-PGF2α, in hibernating brown bears during winter compared to active individuals in summer (Supplementary Table S5). Interestingly, plasma levels of 7-ketocholesterol and 11ß-PGF2α correlated negatively with HDL 12-9 nm and positively with proportions of HDL3c small particles across all individual brown bears (Fig. 5, see legend for specific statistics and regression coefficients). Further, the level of 11ß-PGF2α was negatively associated with proportions of HDL2b, known to be cardioprotective, but not with that of 7-ketocholesterol (Fig. 5).

### Blood antioxidant defenses and oxidative stress markers

**Figure 6:**
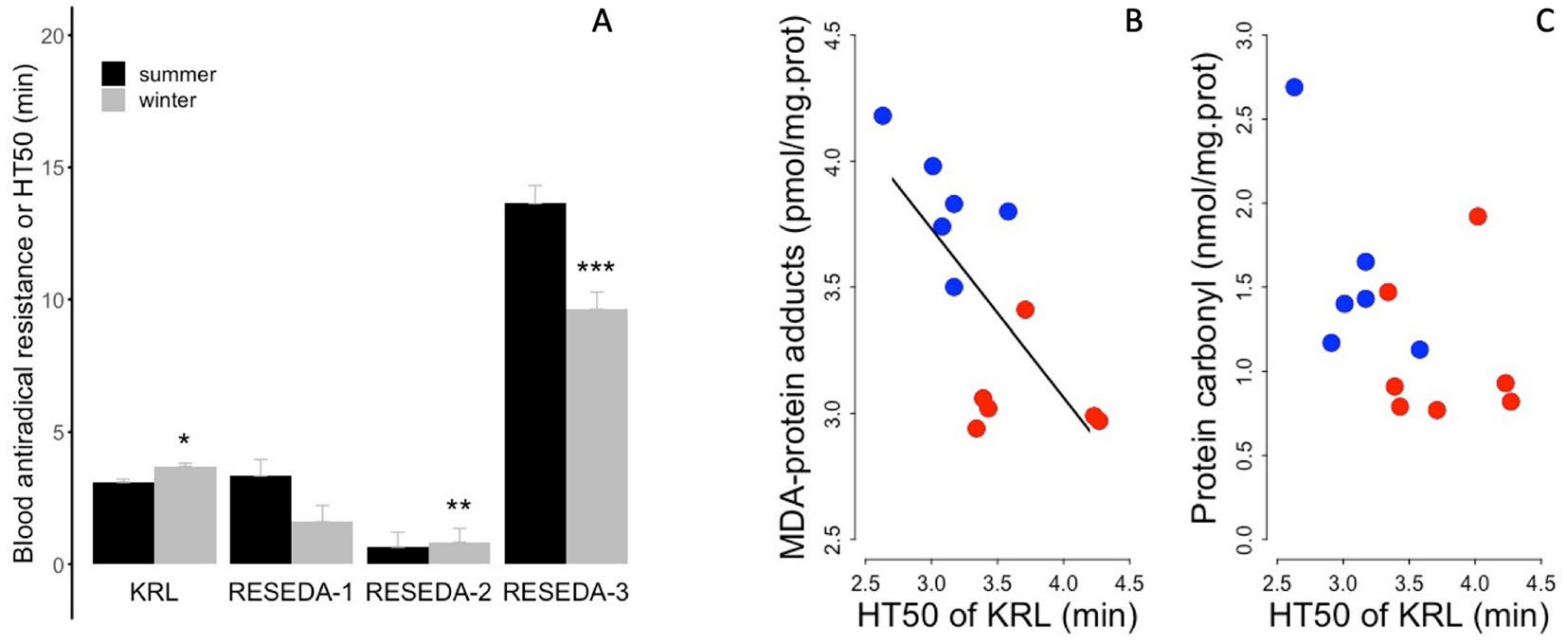
Antiradical resistance of the whole blood according to season and muscle oxidative damage as a function of whole blood antioxidant defenses. (**A**) Half-time of the hemolysis of red blood cells (‘HT50’) from the KRL test without (‘KRL’) or with application of restriction enzymes, *i.e.*, antiradical defense reserves (‘RESEDA-1’, ‘RESEDA-2’, ‘RESEDA-3’) of summer active (‘summer’) and winter hibernating (‘winter’) brown bears. In contrast to summer active individuals, winter hibernating bears were all torpid as indicated by their body temperature during hibernation (see Table 1). Error bars represent standard errors. Winter levels differing significantly from their respective summer level are denoted by a subscript (*p < 0.05, **p < 0.01, ***p < 0.001). Markers of (**B**) muscle lipid peroxidation, ‘Malondialdehyde (MDA)-protein adducts’, and (**C**) muscle protein oxidation, ‘Protein carbonyls’, are represented as function of HT50 of the KRL test from summer active (blue dots) and winter hibernating (red dots) brown bears. Regression statistics from linear mixed-effects models: (MDA) R^2^ = 0.49, t = − 3.23, p = 0.02; (Prot. Carb) R^2^ = 0.25, t = − 2.03, p = 0.09.

The overall antioxidant capacity of blood was altered according to the seasonal status of the brown bear. We found a significant 20% greater half-time of hemolysis (HT50; KRL test), *i.e.,* a higher antiradical resistance of red blood cells during winter hibernation (Fig. 6A, Supplementary Table S6). Interestingly, RESEDA-2, the second contingent of antiradical defense reserves corresponding to sulfatases, showed a significant increase of 27%, which was paralleled by a 40% reduction of RESEDA-3, composed of glucuronidases, in winter hibernating bears compared to summer individuals (Fig. 6A, Supplementary Table S6). We observed no significant seasonal difference in RESEDA-1, *i.e.,* glucosidases, between seasons.

We found that antiradical resistance of red blood cells (half-time of hemolysis of the KRL) was negatively correlated with a marker of lipid peroxidation, *i.e.,* malondialdehyde (MDA)-protein adducts, but was not significantly associated with a marker of protein oxidation, i.e., protein carbonyls (Fig. 6B,C, read legend for specific statistics and regression coefficients). Muscle level of MDA-protein adducts was significantly lower in brown bears during winter hibernation *vs.* summer active season (Supplementary Table S7). Further, we observed a trend to lower level of protein carbonyls in hibernating bears compared to summer individuals (Supplementary Table S7). In contrast, plasma level of MDA-protein adducts, but not that of protein carbonyls, was significantly higher by 30% in winter hibernating brown bears compared to summer active individuals (Supplementary Table S7).

## Discussion

**Figure 7:**
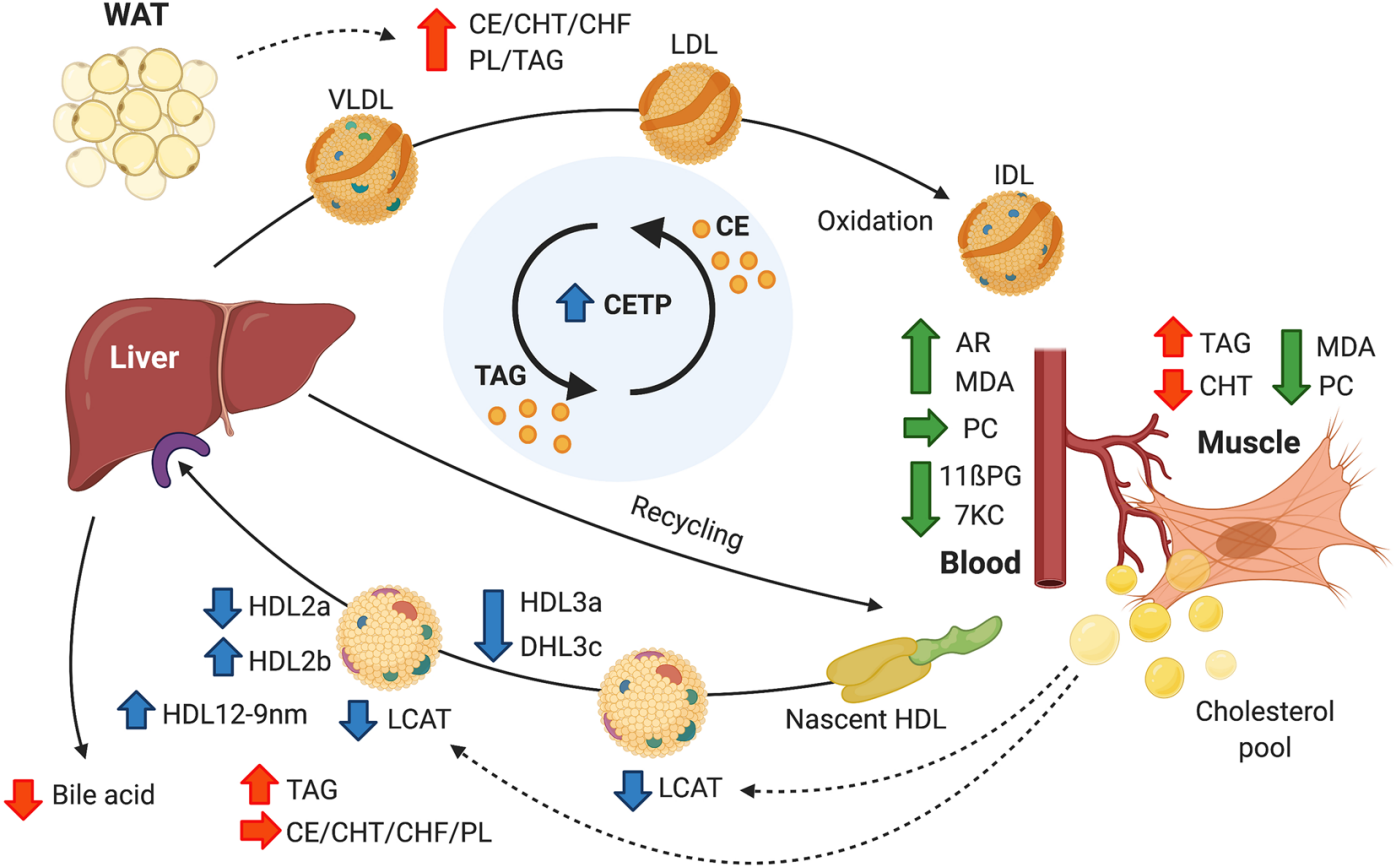
Futile cycles and re-esterification of lipids in hibernating brown bears during winter. Lipids are released from the white adipose tissue (‘WAT’) and mobilized through the bloodstream for oxidation at peripheral tissues. The increased enzymatic activity of the cholesteryl ester transfer protein (‘CETP’) in the plasma allows hibernating bears to recycle cholesterol via exchanges of cholesteryl-esters (‘CE’) and triacylglycerols (‘TAG’) between lipoproteins. ‘CE’ is transferred from high-density lipoproteins (‘HDL’) to very low- (‘VLDL’), low- (‘LDL’), and intermediate-density lipoproteins (‘IDL’), which fuel peripheral tissues, e.g., skeletal muscle (‘Muscle’). Further, a reduced rate of cholesterol synthesis at peripheral tissues, along with a lower activity level of the lipoprotein-bound lecithin-cholesterol acyltransferase (‘LCAT’), lead to maintain constant cholesterol contents in HDL, while increasing HDL size and reducing numbers of HDL particles 2a (‘HDL2a’), 3a (‘HDL3a’), and 3c (‘HDL3c’), at the exception of HDL subunits 2b (‘HDL2b’) known to be cardioprotective that increases. Also, the pro-inflammatory and pro-oxidative properties of the blood is reduced in winter hibernating brown bears as indicated by lowered plasma levels of the oxysterol, 7-Ketocholesterol (‘7KC’), and the isoprostane, 11ß-Prostaglandin F2α (‘11ßPG’), both indexes for atherosclerosis and cardiovascular risks. Because of the unique fast of hibernating bears, excretion of excess cholesterol through bile acid is not possible, leading to increased level of total (‘CHT’) and free cholesterol (‘CHF’), CE, TAG and phospholipids (‘PL’) in plasma, but not of muscle CHT level which decreases. Along with these higher lipid fluxes, blood antiradical resistance (‘AR’) is increased, dampening and/or reducing oxidative damages linked to lipid auto-oxidation, e.g., MDA-protein adducts (‘MDA’) or protein-carbonyls (‘PC’), in plasma and peripheral tissues, including skeletal muscles. Arrows indicate the directions of the changes. Colors of the arrows represent different components concerned by the changes: red for the lipid categories, blue for enzymatic activities and lipoproteins, and green for the players involved in inflammatory and pro-oxidative processes. Created with https://biorender.com.

The present study investigates and highlights (as illustrated in Fig. 7) the unique ability of a large hibernator, the brown bear, which does not excrete excess cholesterol (by defecating and urinating) during hibernation, to challenge the handling of large fluxes of lipids while fasting during several months in winter. Here we report that bears display a plasma lipid profile, i.e., increased plasma levels of NEFA and TAG (Fig. 1), typical of a phase 2 fast with lipolysis providing the main source of energy during winter. Such an increase in lipid mobilization reflects the occurrence of a switch in substrate metabolism toward the sparing of muscle proteins during hibernation (for review, see^68^). Our results further highlight that hibernating bears handle fluxes of TAGs and CHT via futile cycles and re-esterification through lipoprotein metabolism. Specifically, the lipid composition of HDL particles remains stable, while solely HDL2b subunit, known to be cardioprotective, increases during hibernation. In addition, hibernating bears increase blood antioxidant capacities and selectively mobilize SFAs, which are less prone to peroxidation than unsaturated fatty acids, to limit oxidative damages associated to higher lipid fluxes.

### Winter fast does not affect the composition of high-density lipoproteins, of which subclass levels decrease in hibernating brown bears

During fasting, fatty acids are continuously released from white adipose tissue for oxidation or recycled by the liver, leading to the efflux of cellular cholesterol pools into plasma and contributing to the rise in cholesterol levels in lipoproteins, notably in HDL^69^. In contrast to fasted rodents and other hibernators, we observed a stable lipid composition of HDL with only a modest increase in TAG concentrations in hibernating brown bears compared to active individuals in summer (Fig. 3A). Such limited alterations of HDL composition in hibernating brown bears suggest the existence of an active process of re-esterification of lipids and cholesterol through a futile cycle during the hibernation of brown bears. This hypothesis is notably supported by higher levels of enzymatic activity of the CETP, *i.e.,* cholesteryl-ester transfer protein, found in the plasma of brown bears of this study during hibernation compared to the active individuals in summer (Fig. 4A,D). Indeed, CETP, also called plasma lipid transfer protein, facilitates the transport of CEs and TAGs between lipoproteins, by collecting TAGs from VLDL or LDL in exchange to CEs from HDL, and vice versa (for review, see^70^). This enzyme is then directly involved in regulating the amount of circulating HDL-cholesterol within the plasma, and lower levels of CETP promote HDL formation. In particular, genetic deficiency in CETP leads in humans to a drastic rise of HDL-cholesterol^71–73^, and it is the most common cause of hyperalphalipoproteinemia, *i.e.,* elevated HDL, in Japanese people^74^. Hyperalphalipoproteinemia is particularly associated with risks of cardiovascular diseases, including coronary artery disease (CAD). Hence, the increased activity of CETP observed in hibernating brown bears likely contributes to a re-esterification of cholesterol and TAGs via a futile cycle between HDL and VLDL/LDL during the winter fast. However, because of a stable, but not reduced, HLD-cholesterol, one could also argue for an increased hepatic uptake of cholesterol in hibernating brown bears during winter. The occurrence of such a process cannot be excluded and would lead, in parallel to a recycling of CEs, to an accumulation of cholesterol into the liver of hibernating brown bears (for overview, see Fig. 7).

Plasma CETP activity is also closely related to HDL subclass distribution. In particular, CETP enhances HDL remodeling from large (> 10 nm) to small (< 8 nm) HDL subclasses, and human CETP deficiency is associated with a predominance of large HDL particles displaying increased capacity to mediate cellular cholesterol efflux^75–77^, a marker of cardiovascular disease risk. Our findings in hibernating brown bears revealed a substantial decrease in proportions of nearly all subclasses of HDL, along with an increase in HDL particle size, as indicated by a higher proportion of HDL subunits of diameter greater than 12.9 nm, *i.e.,* HDL 12-9 nm (Fig. 3B,C). Interestingly, plasma proportion of large HDL2b particles was increased and that of small HDL3c subunits was reduced in hibernating brown bears during winter compared to the summer bears phenotype. CAD severity in humans is known to be positively associated with the levels of large VLDL and small HDL particles, such as HDL3c, across 158 men^78^. Further, large HDL2b particles have known specific effects for cardiovascular protection (for review see^79^). Freedman and colleagues^78^ also reported that increased levels of both large (10 to 13 nm) HDL, *i.e.,* HDL2b, and intermediate (8.2 to 10 nm) HDL, *i.e.,* HDL2a, are associated with reduced CAD severity in humans. In hibernating bears, together with the increased proportion of HDL2b particles and decreased proportion of HDL3c, our findings of reduced levels of 7-ketocholesterol (oxysterol) and 11ß-PGF2α (isoprostane), metabolites with inflammatory and pro-oxidative actions promoting atherosclerosis and cardiovascular diseases^45,46^, and their negative associations with HDL particles size including the large HLD2b subunit (Fig. 5), suggest a cardiovascular protective state. This is in accordance with the fact that hibernators are particularly resistant to cardiovascular malfunctions and sudden cardiac arrest during the torpor state (for reviews, see^80–82^), which is especially true for brown bears during winter hibernation^83–86^. Adaptations of lipoprotein metabolism, including the maintenance of ‘healthy’ large HDL-cholesterol, would likely contribute to the outstanding ability of bears to avoid cardiovascular dysfunctions during the hibernating state in winter.

Taken together, our results suggest the existence of processes of re-esterification via a futile cycle or of re-uptake of lipids, especially of cholesterol, by the liver in brown bears during their hibernation in winter. In particular, the conversion of cholesterol to bile acid by the liver is nearly fully arrested, as supported by transcriptional suppression of genes involved in cholesterol metabolism in liver of hibernating American black bears^87^. Hence, the constant supply of fatty acids to fuel hibernation and the inability of hibernating bears to excrete excess cholesterol during winter imply additional specific mechanisms at the synthesis level and/or release rate from peripheral tissues.

### Tight control of circulating amounts of cholesterol during the hibernation in brown bears

Within the organism, plasma concentration of cholesterol depends on the balance between fluxes of cholesterol input, via dietary absorption and synthesis/release by tissues, into and excretion from the body. Because fat-storing hibernators, including brown bears, do not eat during winter, the hibernation eliminates dietary cholesterol intake, making de novo synthesis the only way for new cholesterol to be added to body tissues. Because hibernating bears do not defecate nor urinate^88,89^, excess cholesterol cannot be excreted during the entire winter. Our findings of a stable concentration of cholesterol in HDL of hibernating brown bears (Fig. 3A) suggest the occurrence of (i) a tight control of cholesterol synthesis at the cellular level, (ii) a reduced rate of release of cholesterol from peripheral tissues, and/or (iii) an increased reuptake of cholesterol by the liver. However, the lower concentrations of CHT in bear muscles (Fig. 2) during hibernation strongly suggest that the synthesis and/or release rates of cholesterol are lowered at peripheral tissues in hibernating bears. Fasting lowers the hepatic and intestinal synthesis of cholesterol in rodents^90,91^, and the cholesterol synthesis rate of fat cells is suppressed by 90% during fasting in rats, *Rattus norvegicus*^92^. Further, the synthesis rate of cholesterol has been found to be slightly inhibited by the presence of serum VLDL or LDL particles in the medium, suggesting the implication of a regulatory feedback mechanism on the cholesterol synthesis rate^93^. Because the synthesis of cholesterol requires the mobilization of energy, a reduction of the cholesterol synthesis rate during hibernation might contribute to the energy saving of hibernating brown bears in winter.

Along with decreased muscle CHT levels, we also found a significantly lower enzymatic activity of LCAT, *i.e.,* lecithin-cholesterol acyltransferase, in brown bears during winter hibernation compared to the summer active state (Fig. 4B,E). This result points toward a reduction of the accumulation rate of cholesterol into HDL of hibernating brown bears during winter. The enzyme LCAT converts plasma CHF into CEs, which are then sequestered into the core of a lipoprotein particle, making new synthetized HDL spherical (for review, see^94^). A decrease of LCAT activity is fully in line with the reduced synthesis or release rates of CEs and would contribute to the reduction of energy needs during the hibernating state. Comparative studies of LCAT activity in the plasma of several mammalian and ectothermic species, known to have lower daily and yearly metabolic rates, have revealed considerable inter- and intra-species variations^95^. In particular, the activity of LCAT was quantitatively and substantially lower in ectothermic species, such as snakes, amphisbaenids, and amphibians, compared to mammalian species^95^. In lizards during prolonged fasting, LCAT activity and lipid levels were reduced in plasma, and plasma lipoproteins had a lower proportion of lecithin to CHNE^95^. These studies clearly suggest significant relationships between LCAT activity level, lipoprotein metabolism, and metabolic rates of individuals. This would particularly be true for hibernating bears that can reduce their metabolism to 25% of basal metabolic rates, despite regulating their T_b_ between 30° and 36 °C during winter hibernation^9^. In humans, deficiency of LCAT is associated with some pathologies, including eye disease or atherosclerosis^96^. In addition, HDL abnormalities associated to chronic kidney failure (CKF) are largely due to marked downregulation of LCAT activity^97–99^. In our study, we found that hibernating brown bears appear to be able to manage large lipids fluxes via modification of lipoprotein metabolism, including substantial reductions of LCAT activity, without developing any pathological state, such as CKF^100^, during winter.

All together, these results support the existence of a tight control of cholesterol synthesis and/or release from peripheral tissues, such as muscles, and a reduced cholesterol back transportation into HDL, to limit excessive accumulation of cholesterol within the body, with a possible retention by the liver, in hibernating brown bears during winter. Because lipids are prone to autoxidation, these strong adaptions of lipid fluxes and lipoprotein metabolism are expected to increase lipid-derived atherogenic components within the organism. Nevertheless, the unique adaptations of bears to long-term fasting suggest a strong resistance of the latter to oxidative stress associated with lipid handling during winter.

### Increased blood antioxidant capacities dampen lipid oxidative damages in hibernating brown bears

Along with a larger mobilization of lipids associated with the winter fast, our results revealed limited increases of oxidative damage related to lipid peroxidation in the plasma of hibernating brown bears compared to that of summer active individuals (Supplementary Table S7, Fig. 7 for overview). In particular, we observed an unaltered level of protein carbonyls along with a ~ 30% increase of MDA-protein adducts levels in hibernating brown bears compared to summer active individuals.

This suggests the existence of significant reserves for antioxidant capacities in the blood of hibernating brown bears during winter (read below for further discussion). Like small heterotherms, hibernating brown bears preferentially mobilize SFAs, less prone to auto-oxidation than unsaturated fatty acids, to fuel energy needs during winter, and retain unsaturated fatty acids in their tissues^18^. Such a selective mobilization of lipids would contribute to limit the increase of oxidative stress damage observed in the plasma of hibernating brown bears during winter. Similarly, the heterothermic grey mouse lemur (*Microcebus murinus*) selectively increases the oxidation of SFAs in winter, while increasing torpor expression in response to food limitation^101^. This increase of SFAs mobilization was associated with no change of oxidative stress levels specific to unsaturated lipids, which were retained within the body tissues. Also, the relict South American marsupial, *Dromiciops gliroides*, shows differential expression of genes orchestrating different metabolic changes, including shift in lipid metabolism, in liver and muscle cells during hibernation^20^. Interestingly, hepatic and muscle transcripts of thioredoxin-interacting protein, a potent antioxidant, were found to be overexpressed in *D. gliroides* during torpor^20^.

Our findings further revealed a greater antiradical blood resistance (KRL test) in hibernating brown bears compared to summer active individuals (Fig. 6A). This increased resistance of the blood to oxidative threats was primarily mediated via the third component of antiradical defense reserves (RESEDA-3) composed of glucuronidases, which decreased substantially in hibernating brown bears during winter (Fig. 6A). This reduced level of RESEDA-3 indicates the mobilization and specific use of antioxidant glucuronides, which are dietary components found notably in berries^102^, on which the brown bears we studied heavily rely upon to increase their body energy reserves during the late summer and autumn prior to hibernation^103–105^. Interestingly, glucuronides can also be converted into sulfates, corresponding to the second component of antiradical defense reserves (RESEDA-2), which increased in hibernating brown bears during winter (Fig. 6A). These variations suggest an indirect contribution of glucuronides, via modulations of the sulfate pool, to the overall increased antioxidant capacities of brown bears during winter hibernation. Prior to and during winter, hibernators are known to increase their antioxidant capacities and defenses (for review, see^106^). For instance, hibernating ground squirrels increase the protein levels of antioxidant enzymes, such as superoxide dismutase (‘SOD’) -1 and SOD2, as a potential mechanism to prevent ROS-mediated damage in white adipose tissue^107^. Moreover, the activity of antioxidant enzymes including SOD2 was reported to be increased in the brown adipose tissue of golden-mantled ground squirrels fed a high linoleic acid (18:2 n-6) diet prior to hibernation^108^. Altogether, these findings indicate that the increased blood antioxidant capacities likely contribute to dampen the oxidative damage associated with the large lipid mobilization in hibernating brown bears during winter.

In this study, we further found a significant negative relationship between blood antiradical resistance and a marker of lipid peroxidation (MDA-protein adducts) in muscles of hibernating brown bears (Fig. 6B). This association suggests a substantial contribution of greater antioxidant capacities of the blood in limiting oxidative damages in peripheral tissues, such as skeletal muscles. We have previously reported upregulations of cytosolic antioxidant molecules and the maintenance of the GSH/GSSG ratio in skeletal muscles of hibernating brown bears^109^. Along with these upregulations of antioxidant mechanisms in bear muscles, the increased antiradical defenses of the blood could also help to dampen the levels of oxidative threats to muscles during hibernation. Interestingly, neither tissue nor systemic indices of oxidative damage or inflammation increased with fasting in post-weaned pups of northern elephant seal (*Mirounga angustirostris*), a species adapted to cope with chronic periods of stress^110^. In this species, an increase in blood antioxidant capacities seems to be sufficient to suppress both systemic and tissue indices of oxidative damage during periods of prolonged fasting. Moreover, the innate immune system, associated with inflammatory processes, was found to be downregulated in brown bears during hibernation^111^. Further, we have recently demonstrated that brown bears also reduced levels of eicosanoids (molecules derived from polyunsaturated fatty acids), including those with pro-inflammatory properties, during hibernation^112^. Taken together, these findings likely help explain the limited increase of oxidative damage specific to lipid auto-oxidation in the blood and muscles of hibernating brown bears, despite the large increase of lipid fluxes during winter.

## Limitations of the study

One limitation of the study resides in the descriptive aspect of this work that includes only two timepoints (i.e., summer and winter) within the year. Hence, ‘season’ is not necessarily the same treatment as ‘hibernation’ or ‘fasting’, and their respective effects are then confounded due to the seasonal aspect of this study. Further, the amount of tissue that is possible to collect on bears in the field is limited; hence we could not perform more than a thorough analysis of lipoprotein dynamics, lipid profile, enzyme activities, and oxidative status, which already corresponds to a significant achievement. This collaborative work constitutes, however, a unique study, because it provides a rather full picture of the metabolism of cholesterol and other lipids in brown bears under free-living conditions. For this reason, no analyses of additional targets of interest, such as transporters of cholesterol or macrophages, could have been carried out. Nevertheless, this work is of major relevance because diets in laboratories or zoos fail to reflect the natural diet selection of free-living animals that constrain hibernation physiology and adaptation. In particular, diet is seasonally variable in bears in Scandinavia, as elsewhere^104,105^. In autumn, brown bears in Scandinavia build up fat reserves by mainly feeding on berries, such as from the *Vaccinum* family, that contribute most (49–81%) of the dietary energy content of the bears^103–105^, and also provide them with a major source of antioxidant molecules that directly impact on their hibernation physiology and performances, notably in regard to lipid metabolism and protein/muscle maintenance during hibernation (for review, see^68^).

## Conclusion

After approximately four months of hibernation, brown bears display a typical plasma lipid profile of a phase 2 fast with lipolysis providing the main source of energy during winter. As described in studies of fasting, lipolysis provides many times more energy than required to sustain life, leading to high contents of all of the lipid categories in low- and medium-density lipoproteins. Our results suggest that hibernating bears handle fluxes of TAGs and cholesterol via futile cycles and re-esterification through lipoprotein metabolism (for overview, see Fig. 7), keeping the lipid composition of HDL particles stable, while increasing the HDL2b subunit, which is known to be cardioprotective. Despite higher lipid fluxes, oxidative damages seem to be limited, if not reduced, due to greater antioxidant capacities of the blood and the selective mobilization of SFAs, which are less prone to peroxidation than unsaturated fatty acids. Hence, hibernating bears appear able to manage large fluxes of TAG and cholesterol without the classical adverse effects associated with prolonged fasting in humans and non-hibernators.

## Supplementary Information

Supplementary Information 1.

Supplementary Information 2.

## Data Availability

The datasets generated during and/or analyzed during the current study are available from the corresponding author on reasonable request.
