# Supplementary material for: Hibernating brown bears are protected against atherogenic dyslipidemia
Source: Sci Rep. 2021 Sep 21;11:18723. doi: 10.1038/s41598-021-98085-7 (PMC8455566; doi:10.1038/s41598-021-98085-7)
Supplement: Supplementary file 1 — Supplementary Information 1. [file 41598_2021_98085_MOESM1_ESM.pdf]

SUPPLEMENTARY METHODS

HIBERNATING BROWN BEARS ARE PROTECTED AGAINST ATHEROGENIC DYSLIPIDEMIA

Sylvain Giroud[1*], Isabelle Chery[2,3], Mathilde Arrivé[2,3], Michel Prost[4], Julie Zumsteg[5], Dimitri Heintz[5] Alina L. Evans[6], Guillemette Gauquelin-Koch[7], Jon M Arnemo[6,8], Jon E. Swenson[9], Etienne Lefai[10], Fabrice Bertile[2,3], Chantal Simon[11#], Stéphane Blanc[2,3#]

[1] Research Institute of Wildlife Ecology, Department of Interdisciplinary Life Sciences, University of Veterinary Medicine, Vienna, Savoyenstraße 1, 1160 Vienna, Austria

[2] University of Strasbourg, 4 rue Blaise Pascal, 67081 Strasbourg, France

[3] CNRS, UMR7178, Institut Pluridisciplinaire Hubert Curien (IPHC), 23 rue du Loess, 67087 Strasbourg, France

[4] SPIRAL Laboratories, 21560 Couternon, France

[5] Plant Imaging & Mass Spectrometry (PIMS), Institute of Plant Molecular Biology, CNRS, University of Strasbourg, 12 rue du Général Zimmer, 67084 Strasbourg, France

[6] Department of Forestry and Wildlife Management, Inland Norway University of Applied Sciences, NO-2480 Koppang, Norway

[7] CNES Paris, 2 Place Maurice Quentin, 75039 Paris Cedex 01, France

[8] Department of Wildlife, Fish and Environmental Studies, Swedish University of Agricultural Sciences, SE-90183 Umeå, Sweden

[9] Faculty of Environmental Sciences and Natural Resource Management, Norwegian University of Life Sciences, PO Box 5003, NO-1432 Ås, Norway

[10] University of Auvergne, INRAE, UNH UMR1019, 63122 Saint-Genès Champanelle, France

[11] CARMEN, INSERM U1060 / University of Lyon / INRA U1235, Oullins, France

[*] Corresponding author: Dr. Sylvain Giroud; Research Institute of Wildlife Ecology, Savoyenstraße 1, 1160 Vienna, Austria; Tel: (+43) 1 250 777 135, Fax: (+43) 1 250 777 94 135; sylvain.giroud@vetmeduni.ac.at

[#] These authors contributed equally to the work.

Determination of plasma and lipoprotein lipid composition

Lipoprotein profiles, *i.e.*, very low-density lipoprotein ('VLDL'), intermediate-density lipoprotein ('IDL'), low-density lipoprotein ('LDL'), and high-density lipoprotein ('HDL'), were determined by Synelvia SAS, France. Lipoproteins from plasma samples were separated by liquid chromatography (Dionex ultimate 3000, Thermofischer scientific) using Size Exclusion Chromatography (SEC) on Superose 6 10/300 GLcolumns (300 x 10 mm, GE Healthcare) with online detection of lipoprotein components[1]. After separation by SEC, an enzymatic derivatization with inline detection was performed using annex pump delivering an enzyme mixture that react specifically with total cholesterol ('CHT') (CHOD-PAP method, kit 87656, Biolabo), free cholesterol ('CHF') (CHOD-PAP method, kit 88656, Biolabo), non-esterified cholesterol (CHOD-PAP method, kit 99656, Biolabo), triacylglycerides (GPO method, kit 87319, Biolabo), phospholipids (PLs) (colorimetric method, kit 99110, Biolabo), and non-esterified fatty acids. Cholesteryl-esters ('CE') levels were calculated as the difference between CHT and CHF levels. The same lipid molecules were also quantified in plasma by using the same methods as for lipid quantification in lipoproteins.

Electrophoretic separation of high-density lipoprotein sub-fractions

Plasma lipoproteins were separated by electrophoresis using non-denaturing polyacrylamide gradient gels[2-4]. Densities were first adjusted by the addition of potassium bromide and lipoprotein fractions were isolated from plasma by sequential ultracentrifugation. Aliquots of the fractions d < l.21 g/mL that contain lipoproteins were subjected to electrophoresis using 1.5-25% polyacrylamide gradient gels (SpiraGel™), according to the manufacturer instructions (LARA SPIRAL, Couternon, France). The apparent diameter of the separated lipoprotein sub-fractions was determined by comparison with a high molecular weight protein calibration kit (Pharmacia) subjected to electrophoresis together with the samples. The distribution profile of LDL and HDL sub-fractions was finally obtained by densitometry scanning. The relative proportions of HDL sub-fraction 2a ('HDL2a'), 2b ('HDL2b'), 3a ('HDL3a'), 3b ('HDL3b'), 3c ('HDL3c') were determined as the ratio of the corresponding areas under the scan curve to the area corresponding to the sum of HDL sub-fractions. We also determined the proportion of HDL subunits with a diameter greater than 12.9 nm ('HDL 12-9nm') and computed the ratio between levels of LDL and HDL subunits ('LDL/HDL').

Quantification of neutral lipids in muscles

We extracted lipids from 1 mg of muscle using a procedure described by Bligh and Dyer[5] in dichloromethane/methanol/water (2.5/2.5/2.1, v/v/v) in the presence of the internal standards: 4 μg stigmasterol, 4 μg cholesteryl heptadecanoate, 8 μg glyceryl trinonadecanoate. The dichloromethane phase was evaporated to dryness and samples were then dissolved in 20 ml ethyl acetate. 1 μl of the lipid extract was analyzed by gas-liquid chromatography on a FOCUS Thermo Electron system using Zebron-1 Phenomenex fused silica capillary columns (5m X 0.32mm i.d, 0.50 μm film thickness)[6]. Oven temperature was programmed from 200°C to 350°C at a rate of 5°C per min and the carrier gas

was hydrogen (0.5 bar). The injector and the detector temperatures were set to 315°C and 345°C, respectively. For each lipid species, the relative quantification was obtained by comparing the area under the peak for the lipid of interest with that from its internal standard.

Assessment of levels of ß-hydroxybutyrate and enzymatic activities

Plasma level of ß-hydroxybutyrate ('ßOHB') was assessed via a specific kit (Kit Enztec TM Fluid beta hydroxybutyric acid #5250). Plasma enzymatic activities were measured via specific assays for cholesteryl-ester transfer protein ('CETP') (CETP Activity Assay Kit Fluorometry Abnova #KA0790), lecithin-cholesterol acyltransferase ('LCAT') (LCAT Activity Assay Kit Merck Millipore #42890), and phospholipid transfer protein ('PLTP') (PLTP Activity Assay Kit Abnova #KA0791).

Measurements of oxysterols and isoprostanes

The levels of three oxysterols, including 7-ketocholesterol, 7α-hydroxycholesterol, and 7ß-hydroxycholesterol, and four isoprostanes, including 8-iso prostaglandin F2α (8-iso PGF2α), 8-iso-15(R) prostaglandin F2α (8-iso -15(R) PGF2α), 11ß-prostaglandin F2α (11ß-PGF2α), and 15(R)-prostaglandin F2α (15(R)-PGF2α), were measured in the blood plasma of winter hibernating and summer active brown bears (N=16/season). To this end, UPLC-MS/MS multiple reaction monitoring (MRM)-based assays were developed, using pure compound standards purchased from Avanti Polar Lipids, Inc. (Alabaster, AL, USA) for oxysterols and from Cayman chemicals (Ann Arbor, MI, USA) for isoprostanes. Methanol, formic acid, and isopropanol have been purchased from ThermoFisher Scientific (Rockford, IL, USA).

After drying of 150 µl of plasma samples using a SpeedVac, resuspension was performed using 150 µl of cold ethyl acetate (Honeywell International Inc., Charlotte, NC, USA) and extracts were homogenized under agitation (1000 rpm) at 5°C during 20 min, then centrifuged (13000 rpm) for 15 min at 4°C to pellet any possible particle prior to mass spectrometry analysis.

For oxysterols, analyses were performed on a Acquity UPLC^{TM} system (Waters, Milford, MA, USA) coupled to a triple quadrupole mass spectrometer (Quattro Premier XE^{TM}; Waters) equipped with an Atmospheric Pressure Photon Ionisation (APPI) source. HPLC mobile phases were methanol 75% in water with 0.1% formic acid (A) and isopropanol 99.99% with 0.01% formic acid (B). Chromatographic separation of samples (5µl) was performed using a Acquity UPLC BEH C8 VanGuard pre-column (130Å, 1.7 µm, 2.1 mm x 5 mm; Waters), and a Acquity UPLC BEH C8 column (130Å, 1.7 µm, 2.1 mm x 100 mm; Waters) operated at 46°C. Elution started with 100% of solvent A at a flow rate of 0.37 ml/min maintained for 2 min; it was followed by a 25-min gradient at 0.3 ml/min to reach 50% of A, then a 3-min gradient at 0.25 ml/min to reach 44% of A, and a last gradient step at 0.2ml/min to reach 100% of B in 8 min, which was afterwards maintained for 3 min. Then another 4-min gradient was applied at 0.37 ml/min to reach 100% of solvent A, which was maintained for 3 min. The mass spectrometer was parametrized as follows: the nebulizer gas flow was set to 50L/h and the desolvatation gas flow to 500L/h; the APPI probe temperature was set to 450°C and the source temperature to 120°C; the capillary voltage was set to 1.5kV. The MRM mode was used based on the selection of the most

abundant ions for quantitative analysis. Absolute quantifications were achieved by comparison of sample signals with dose–response curves established with pure standards. The MS/MS MRM transitions that we monitored were as follows: 401.3>109.0 for 7-ketocholesterol, 367.5>159.2 for 7α-hydroxycholesterol, and 385.35>105 for 7ß-hydroxycholesterol. Data acquisition and analysis were performed with MassLynx software (v4.1; Waters).

For isoprostanes, analyses were performed on a Dionex UltiMate 3000 UHPLC system (ThermoFisher Scientific) coupled to triple quadrupole mass spectrometer (EvoQ EliteTM, Bruker Daltonik GmbH, Bremen, Germany) equipped with an electrospray ionization source. HPLC mobile phases were water (C) and methanol (D), both with 0.1% formic acid. Chromatographic separation of samples (5µl) was performed using a Acquity UPLC HSS T3 VanGuard pre-column (100Å, 1.8 µm, 2.1 mm x 5 mm; Waters), then separation was achieved using a Acquity UPLC HSS T3 Column (100Å, 1.8 µm, 2.1 mm x 100 mm; Waters) operated at 40°C. Elution was performed at a flow-rate of 0.40 mL/min; it started with 90% of solvent C, then a linear gradient was applied to reach 60% of D in 5 min, followed by a 25-min convex gradient to reach 100% of D, which was maintained for 3 min. Return to initial conditions was achieved in 1 min and it was maintained for 1 min. The mass spectrometer was operated in negative mode and parametrized as follows: the nebulizer gas flow was set to 20 L/h, and the desolvation gas flow to 20 L/h; the source temperature was set to 350°C and the interface temperature to 300 °C; the capillary voltage was set to 4 kV. Low mass and high mass resolution were 2 for the first mass analyzer and 2 for the second. The MRM mode was used based on the selection of the most abundant ions for quantitative analysis. Absolute quantifications were achieved by comparison of sample signals with dose–response curves established with pure standards. The MS/MS MRM transitions that we monitored were as follows: 353.1>193.1 and 353.1>309.2 for 8-iso PGF2α, 353.1>193.1 and 353.1>309.2 for 11ß-PGF2α, 353.1>193.1 and 353.1>309.2 for 15(R)-PGF2α, 353.1>193.1 and 353.1>309.2 for 8-iso -15(R) PGF2α. The instruments were controlled by Compass Hystar (v4.1 SR1, Bruker) and MS Workstation (v8, Bruker). Data analysis was performed with MS Data Review software (v8.2.1; Bruker Daltonik GmbH).

Determination of blood antiradical resistance and associated antiradical defense reserves

The overall antioxidant capacity plasma samples was assessed with the biological KRL/RESEDA™ test (Kirial International/Laboratories Spiral, Couternon, France – M.Prost-Spiral patent)[7-10]. In the KRL test, carried out in 96-well microplates, a control blood (defibrinated horse blood) was subjected to free radical attack under controlled and standardized conditions (37°C, orbital shaking), in the presence of bear plasma sample. Enzymatic and chemical antioxidant systems protect the integrity of the blood cells until hemolysis. Measuring the decrease in absorbance (620 nm) allows the monitoring of the progressive disappearance of the cells. The resistance of blood cells against free radical attack is expressed as the time necessary to lyse 50% of the blood cells. Hence, we determined the half-time of the hemolysis of red blood cells (‘HT50’, expressed in min) exposed to a controlled free radical attack.

The bear plasma antiradical efficacy is then expressed as a percentage increase of the overall antiradical defense potential of the control blood.

The antiradical defense reserves (RESEDA) test is based on the same principle of the KRL test with a step of releasing biologically active and potentially antioxidant molecules in reserve as RESEDA-1 (glucosides), RESEDA-2 (sulphates), and RESEDA-3 (glucuronides)[8,11].

Determination of oxidative stress markers

The contents of malondialdhyde (MDA)-protein adducts and protein-carbonyls were measured in both plasma and skeletal muscle of brown bears using commercial ELISA kits (#STA-332 and #STA-315, respectively) from Cell Biolabs (San Diego, CA, USA).

REFERENCES

1 Usui, S., Hara, Y., Hosaki, S. & Okazaki, M. A new on-line dual enzymatic method for simultaneous quantification of cholesterol and triglycerides in lipoproteins by HPLC. *J Lipid. Res.* **43**, 805-814, 10.1016/s0022-2275(20)30123-1 (2002).

2 Blanche, P. J., Gong, E. L., Forte, T. M. & Nichols, A. V. Characterization of human high-density lipoproteins by gradient gel electrophoresis. *Biochim. Biophys.* **665**, 408-419, 10.1016/0005-2760(81)90253-8 (1981).

3 Gambert, P., Bouzerand-Gambert, C., Athias, A., Farnier, M. & Lallemant, C. Human low density lipoprotein subfractions separated by gradient gel electrophoresis: composition, distribution, and alterations induced by cholesteryl ester transfer protein. *J Lipid. Res.* **31**, 1199-1210, 10.1016/s0022-2275(20)42628-8 (1990).

4 Zeller, M., Masson, D., Farnier, M., Lorgis, L., Deckert, V., Pais de Barros, J.-P. *et al.* High Serum Cholesteryl Ester Transfer Rates and Small High-Density Lipoproteins Are Associated With Young Age in Patients With Acute Myocardial Infarction. *J Am. Coll. Cardiol.* **50**, 1948-1955, 10.1016/j.jacc.2007.06.052 (2007).

5 Bligh, E. G. & Dyer, W. J. A rapid method of total lipid extraction and purification. *Can. J Biochem. Phys.* **37**, 911-917, 10.1139/o59-099 (1959).

6 Barrans, A., Collet, X., Barbaras, R., Jaspard, B., Manent, J., Vieu, C. *et al.* Hepatic Lipase Induces the Formation of Pre-β_1 High Density Lipoprotein (HDL) from Triacylglycerol-rich HDL_2. A study comparing liver perfusion to *in vitro* incubation with lipases. *J Biol. Chem.* **269**, 11572-11577 (1994).

7 Prost, M. Process for the determination by means of free radicals of the antioxidant properties of a living organism or a potentially aggressive age. U.S. patent 20060234329.A1 (1989).

8 Prost, M. Method for Determining the Antiradical Defense Potential and Use Thereof, in Particular in Veterinary and Human Preventive Therapeutics. U.S. patent 20060234329.A1 (2003).

9 Lesgards, J. F., Durand, P., Lassarre, M., Stocker, P., Lesgards, G., Lanteaume, A. *et al.* Assessment of lifestyle effects on the overall antioxidant capacity of healthy subjects. *Environ. Health Perspect.* **110**, 479-486, 10.1289/ehp.02110479 (2002).

10 Rossi, R., Pastorelli, G. & Corino, C. Application of KRL test to assess total antioxidant activity in pigs: Sensitivity to dietary antioxidants. *Res. Vet. Sci.* **94**, 372-377, 10.1016/j.rvsc.2012.08.005 (2013).

11 Cases, J., Romain, C., Marín-Pagán, C., Chung, L. H., Rubio-Pérez, J. M., Laurent, C. *et al.* Supplementation with a Polyphenol-Rich Extract, PerfLoad, Improves Physical Performance during High-Intensity Exercise: A Randomized, Double Blind, Crossover Trial. *Nutrients* **9**, 421, 10.3390/nu9040421 (2017).
