# Supplementary material for: Hibernating brown bears are protected against atherogenic dyslipidemia
Source: Sci Rep. 2021 Sep 21;11:18723. doi: 10.1038/s41598-021-98085-7 (PMC8455566; doi:10.1038/s41598-021-98085-7)
Supplement: Supplementary file 2 — Supplementary Information 2. [file 41598_2021_98085_MOESM2_ESM.pdf]

SUPPLEMENTARY TABLES

HIBERNATING BROWN BEARS ARE PROTECTED AGAINST ATHEROGENIC DYSLIPIDEMIA

Sylvain Giroud[1*], Isabelle Chery[2,3], Mathilde Arrivé[2,3], Michel Prost[4], Julie Zumsteg[5], Dimitri Heintz[5] Alina L. Evans[6], Guillemette Gauquelin-Koch[7], Jon M Arnemo[6,8], Jon E. Swenson[9], Etienne Lefai[10], Fabrice Bertile[2,3], Chantal Simon[11#], Stéphane Blanc[2,3#]

[1] Research Institute of Wildlife Ecology, Department of Interdisciplinary Life Sciences, University of Veterinary Medicine, Vienna, Savoyenstraße 1, 1160 Vienna, Austria

[2] University of Strasbourg, 4 rue Blaise Pascal, 67081 Strasbourg, France

[3] CNRS, UMR7178, Institut Pluridisciplinaire Hubert Curien (IPHC), 23 rue du Loess, 67087 Strasbourg, France

[4] SPIRAL Laboratories, 21560 Couternon, France

[5] Plant Imaging & Mass Spectrometry (PIMS), Institute of Plant Molecular Biology, CNRS, University of Strasbourg, 12 rue du Général Zimmer, 67084 Strasbourg, France

[6] Department of Forestry and Wildlife Management, Inland Norway University of Applied Sciences, NO-2480 Koppang, Norway

[7] CNES Paris, 2 Place Maurice Quentin, 75039 Paris Cedex 01, France

[8] Department of Wildlife, Fish and Environmental Studies, Swedish University of Agricultural Sciences, SE-90183 Umeå, Sweden

[9] Faculty of Environmental Sciences and Natural Resource Management, Norwegian University of Life Sciences, PO Box 5003, NO-1432 Ås, Norway

[10] University of Auvergne, INRAE, UNH UMR1019, 63122 Saint-Genès Champanelle, France

[11] CARMEN, INSERM U1060 / University of Lyon / INRA U1235, Oullins, France

[*] Corresponding author: Dr. Sylvain Giroud; Research Institute of Wildlife Ecology, Savoyenstraße 1, 1160 Vienna, Austria; Tel: (+43) 1 250 777 135, Fax: (+43) 1 250 777 94 135; sylvain.giroud@vetmeduni.ac.at

[#] These authors contributed equally to the work.

Short title: Lipoprotein metabolism in brown bears

Supplementary Table S1: Means, standard errors ('SE') and p-values of log-transformed differences in plasma concentrations or differences in muscle proportions of main categories of lipids ('variables') between winter and summer phenotypes in brown bears. For both plasma and muscle, lipid categories are non-esterified cholesterol ('CHNE'), total cholesterol ('CHT'), and triacylglycerols ('TAG'). Only for plasma, lipid categories further include non-esterified fatty acids ('NEFA') and phospholipids ('PL'), as well as ß-hydroxybutyrate (ßOH). Significant p-values are highlighted in bold.

		**Winter – summer differences**		
**Tissues**	**Variables**	**Means**	**SE**	**P-values**
**Plasma**				
	CHNE	0.42	0.07	**<0.001**
	CHT	0.34	0.06	**<0.001**
	TAG	0.72	0.12	**<0.001**
	NEFA	1.27	0.30	**0.003**
	PL	0.21	0.06	**0.011**
	ßOH	6.03	0.50	**<0.001**
**Muscle**				
	CHNE	-0.76	0.55	0.209
	CHT	-30.36	7.98	**0.002**
	TAG	31.06	8.28	**0.002**

Supplementary Table S2: Means, standard errors ('SE') and p-values of log-transformed differences in plasma concentrations of different lipid categories ('variables') for the main classes of lipoproteins between winter and summer phenotypes in brown bears. Lipoprotein classes correspond to high-density lipoproteins ('HDL'), intermediate-density lipoproteins ('IDL'), low-density lipoproteins ('LDL'), and very low-density lipoproteins ('VLDL'). Lipid categories include non-esterified cholesterol ('CHNE'), free cholesterol ('CHF'), total cholesterol ('CHT'), phospholipids ('PL') and triacylglycerols ('TAG'). Significant p-values are highlighted in bold.

		**Winter – summer differences**		
**Classes**	**Variables**	**Means**	**SE**	**P-values**
**HDL**				
	CHNE	-0.05	0.09	0.587
	CHF	-0.12	0.10	0.257
	CHT	-0.07	0.09	0.454
	PL	-0.19	0.09	0.066
	TAG	0.34	0.07	**0.002**
**IDL**				
	CHNE	0.96	0.15	**<0.001**
	CHF	1.09	0.12	**<0.001**
	CHT	1.03	0.13	**<0.001**
	PL	0.95	0.13	**<0.001**
	TAG	1.17	0.13	**<0.001**
**LDL**				
	CHNE	0.49	0.10	**0.002**
	CHF	0.37	0.09	**0.005**
	CHT	0.41	0.08	**<0.001**
	PL	0.34	0.08	**0.005**
	TAG	0.42	0.10	**0.005**
**VLDL**				
	CHNE	2.05	0.17	**<0.001**
	CHF	2.32	0.23	**<0.001**
	CHT	2.18	0.20	**<0.001**
	PL	2.15	0.18	**<0.001**
	TAG	2.26	0.21	**<0.001**

Supplementary Table S3: Means, standard errors ('SE') and p-values of log-transformed differences in plasma proportions of subclasses and sizes of lipoproteins ('variables') between winter and summer phenotypes in brown bears. Lipoprotein subclasses correspond to high-density lipoproteins 2a ('HDL2a'), 2b ('HDL2b'), 3a ('HDL3a'), 3b ('HDL3b'), and 3c ('HDL3c'). Lipoprotein size and ratio includes HDL subunits of diameter greater than 12.9 nm ('HDL 12.9nm') and the ratio between levels of LDL and HDL ('LDL/HDL'). Significant p-values are highlighted in bold.

	Winter – summer differences		
**Variables**	**Means**	**SE**	**P-values**
**HDL2a**	-0.14	0.03	**0.002**
**HDL2b**	0.31	0.13	**0.047**
**HDL3a**	-0.21	0.04	**<0.001**
**HDL3b**	-0.17	0.07	0.081
**HDL3c**	-0.41	0.07	**0.002**
**HDL 12-9nm**	1.53	0.11	**<0.001**
**LDL/HDL**	0.12	0.06	0.073

Supplementary Table S4: Means, standard errors ('SE') and p-values of log-transformed differences in lipid enzymatic activities between winter and summer phenotypes in brown bears. Enzymes correspond to cholesteryl ester transfer protein ('CETP'), lecithin-cholesterol acyltransferase ('LCAT'), and phospholipid transfer protein ('PLTP'). Significant p-values are highlighted in bold.

	**Winter – summer differences**		
**Variables**	**Means**	**SE**	**P-values**
**CETP**	0.30	0.09	**0.008**
**LCAT**	-0.51	0.04	**<0.001**
**PLTP**	-0.06	0.13	0.632

Supplementary Table S5: Means, standard errors ('SE') and p-values of Box-Cox transformed differences in levels of 7-Ketocholesterol (oxysterol) and of 11ß-Prostaglandin (isoprostane) between winter hibernating and summer active brown bears. Significant p-values are highlighted in bold.

	Winter – summer differences		
**Variables**	**Means**	**SE**	**P-values**
**7-Ketocholesterol**	-8.36	2.02	**0.003**
**11ß-Prostaglandin**	-0.26	0.20	**0.006**

Supplementary Table S6: Means, standard errors ('SE') and p-values of log-transformed differences of the half-time of the hemolysis of red blood cells ('HT50') from the KRL test without ('KRL') or with application of restriction enzymes, *i.e.*, antiradical defense reserves ('RESEDA-1', 'RESEDA-2', 'RESEDA-3'), between winter and summer phenotypes in brown bears. Significant p-values are highlighted in bold.

	**Winter – summer differences**		
**Variables**	**Means**	**SE**	**P-values**
**KRL**	0.18	0.06	**0.020**
**RESEDA-1**	-0.55	0.27	0.107
**RESEDA-2**	-0.70	0.15	**0.012**
**RESEDA-3**	-0.36	0.07	**<0.001**

Supplementary Table S7: Means, standard errors ('SE') and p-values of differences in plasma or muscle levels of markers of 'Malondialdehyde (MDA)-protein adducts' (lipid peroxidation) and of 'Protein carbonyls' (protein oxidation) between winter and summer phenotypes in brown bears. Significant p-values are highlighted in bold.

		Winter – summer differences		
**Tissues**	**Variables**	**Means**	**SE**	**P-values**
**Plasma**				
	MDA-protein adducts	16.54	2.90	**0.002**
	Protein carbonyls	-0.03	0.01	0.119
**Muscle**				
	MDA-protein adducts	-0.77	0.09	**0.001**
	Protein carbonyls	-1.36	0.70	0.108
